# The English Courts Deal with Substitution

**Published:** 1904-08

**Authors:** 


### The English Courts Deal With Substitution.

FOLLOWING closely upon the heels of the successful fight against medical substitution in New York City wherein the proprietors of pepto-mangan, Gude, secured a permanent injunction against a big department store restraining it from exposing any preparation in any way similar to their protected article, word comes from London of the end of a substitution case which will take its place in medico-legal history as something of a cause celebre, because of the high standing of the representative and distinguished professional men who appeared for the plaintiffs, and because the victory over substitution was so sweeping.

Burroughs-Wellcome & Company have been doing business in England for a great many years and their medical products have become known for accuracy, purity and standard strength. The tablet or compressed pill form of drugs which they fathered were some years ago given the trade name of "tabloids," and as such were advertised to the profession. This "carried" name was registered as a trade-mark under British law. Tabloids became synonomous with honesty in drugs and accuracy in dosage and were specified in prescriptions. Other manufacturers of tablet drugs made no attempt to infringe openly, but many large drug firms did a fine business in substitution. When tabloids were written and the Burroughs-Wellcome make specified, it was the custom of substituters to dispense a similar looking tablet of an entirely different make.

The chief offenders were Thompson and Capper, who own many drug stores, and they were brought into court before Mr. Justice Byrne. It was merely a business case in the beginning and no one apparently took much interest in it until Burroughs-Wellcome & Company began to call witnesses to prove the value of tabloids as a distinct product. Then the drug trade and the medical profession and the lawyers and the reading public generally sat up and took a lively interest in the case, for among the witnesses who appeared opposed to substitution were such men as Sir Francis Laking, surgeon to King Edward; Dr. A. Pearson

Luff, scientific expert to the British Home office; Sir Patrick Manson, medical adviser to the British Colonial office; Prof. Attfield, professor of chemistry of the Pharmaceutical Society of Great Britain and editor of the British Pharmacopeia; Prof. Donald Macalister, of Cambridge; Sir R. Douglas Powell, physician to King Edward; Sir Andrew Critchett, oculist to the King; Sir William Thompson, surgeon to the King in Ireland, and Mr. Reginald Harrison, who is so well known in this country, and who is a member of the American Urologic Association.

These men and many others testified that when they prescribed tabloids they wrote with the understanding that the Burroughs-Wellcome product would be dispensed. Then by the witnesses of Thompson and Capper it was shown that there had been practised substitution of the rankest and most bare-faced description, an inferior and less-certain manufacture of tablet having been substituted. Thompson and Capper in an effort to block the inevitable injunction asked to have the word tabloid removed from the trade-mark register, and this was made a part of the case. Mr. Justice Byrne gave judgment against the substituting firm by dismissing the motion to remove the word tabloid; by granting an injunction against Thompson and Capper, and ordering them to pay damages and costs and also the costs of the comptroller of trade marks. An appeal was taken to the supreme court of judicature, which is equivalent to our court of appeals where, after hearing the arguments and reviewing the evidence, the appeal was dismissed and Thompson and Capper were assessed all the costs of both sides in the appeal.

The outcome of this case is more widespread in England than the recent case in New York, for whereas the New York decision affects only the particular dry goods firm proceeded against, the English decision covers the entire kingdom. The Thompson and Capper people did a good business in substitution as shown by the evidence, and the judgment entered against them will have the effect of pretty generally killing the evil in England for all time to come.

---

Elsewhere in this issue we publish a letter from General De Witt, secretary of the Walter Reed Memorial Association, announcing the incorporation of that body and the readiness of the executive committee to receive contributions. The object is a worthy one and every American physician should feel proud of the achievements of this distinguished physician, who accomplished so much in staying the progress of yellow fever. Major Reed's name will be forever linked with the wiping out of this terrible malady in Havana, which for centuries had been a menace to the lives and health of the people of the United States.

---

New York City's progress in facilities for medical and surgical training under the best conditions has been marvelous. New hospitals and additions to hospitals go up with almost magical speed, and from every state in the Union come novices who have made up their minds to devote their lives to the relief of suffering and the benefit of their fellow beings. Additional clinical buildings and laboratories besides the many already in use are soon to be constructed.

---

In Germany, when a person breaks down with consumption, he is sent to a government sanatorium, where he is kept until he recovers or dies. In the mean time his family receives a weekly pension from a fund to which the patient himself contributed when he was in good health. By this means the risk of spreading the disease is avoided.

---

The court of appeals has rejected the appeal of Antonius, the boy wonder (aged 40), from the sentence of nine months in the penitentiary for swindling sick persons by a fake healing scheme.

Weichers was on bail for a year, but is now in the Erie County Penitentiary serving the time for which he was sentenced. It was said that he was in New York until recently.

---

At the annual convocation of the University of the State of New York, held at Albany, June 27-28, 1904, Dr. Albert Vander Veer, a regent of the university, discussed the subject of higher education as related to medical schools under the following title: "Should the regents register college courses as the equivalent of the first year in a medical school?"

Dr. Vander Veer favored the teaching of certain medical subjects in undergraduate colleges, such as primary anatomy, histology and medical botany, thus permitting advance work in these subjects in the first year in the medical colleges. He argued in favor of a seven years' course (academic and medical combined), and expressed the belief that a definite arrangement of this question was approaching. He believed that encouragement should be given to the seven years' plan, for without it, he declared, there was danger that young men would go directly from the high school to medical colleges, a danger which, he said, should be avoided. He read a large number of opinions of varying nature on the question, outlined a possible curriculum for first year medical work in colleges and closed by declaring that while independent colleges might find it difficult, nevertheless universities having medical departments could arrange for such courses. He believed that the independent colleges would also find it possible on the basis suggested by the regents.

In his address in opening the convocation the Chancellor Hon. Whitelaw Reid said: "If the reorganised board of regents and the new commissioner of education understand themselves and each other and their opportunity, they are firmly resolved that their whole province is a realm in which politics shall never enter."

This is a wise beginning and it is hoped and believed that such a wholesome sentiment always will prevail throughout the management of our educational affairs.

---

Mr. Justice Fitzgerald, of the supreme court in New York, heard arguments in relation to the consolidation of the State Medical Society and the State Medical Association, as provided by chapter 1, laws of the state of New York, 1904.

The Onondaga County Medical Association was the only organisation which opposed granting the order of merger, though a few scattering individuals are antagonising the scheme of medical unity. Mr. Howard Van Sinderen, of New York, appeared for both state organisations in favor of the order, and in the course of his argument said that more than 5,600 out of 5,700 members of the county medical societies were in favor of the proposed plan of amalgamation, as were also more than 1,600 out of 1,767 physicians belonging to the association.

The opposition, consisting of the Onondaga County Medical Association and a few individuals of other associations, was represented by Willard A. Glann, Esq., of Syracuse. Judge Fitzgerald reserved his decision.

---

Vitality of germs of diphtheria for a long time, has been demonstrated by the report of a health officer in Mecosta county to the secretary of the Michigan State Board of Health, who states that twenty years ago, Mrs. T. lost a daughter by death from diphtheria, and then some of the girl's clothing was put away in a chest and nailed up. The chest was not disturbed until this spring, when the mother, 75 years of age, opened it and looked over the clothing, soon after which she was taken sick with diphtheria and died, June 17, 1904. The health officer believes she contracted the disease from the clothing, infected twenty years ago.

